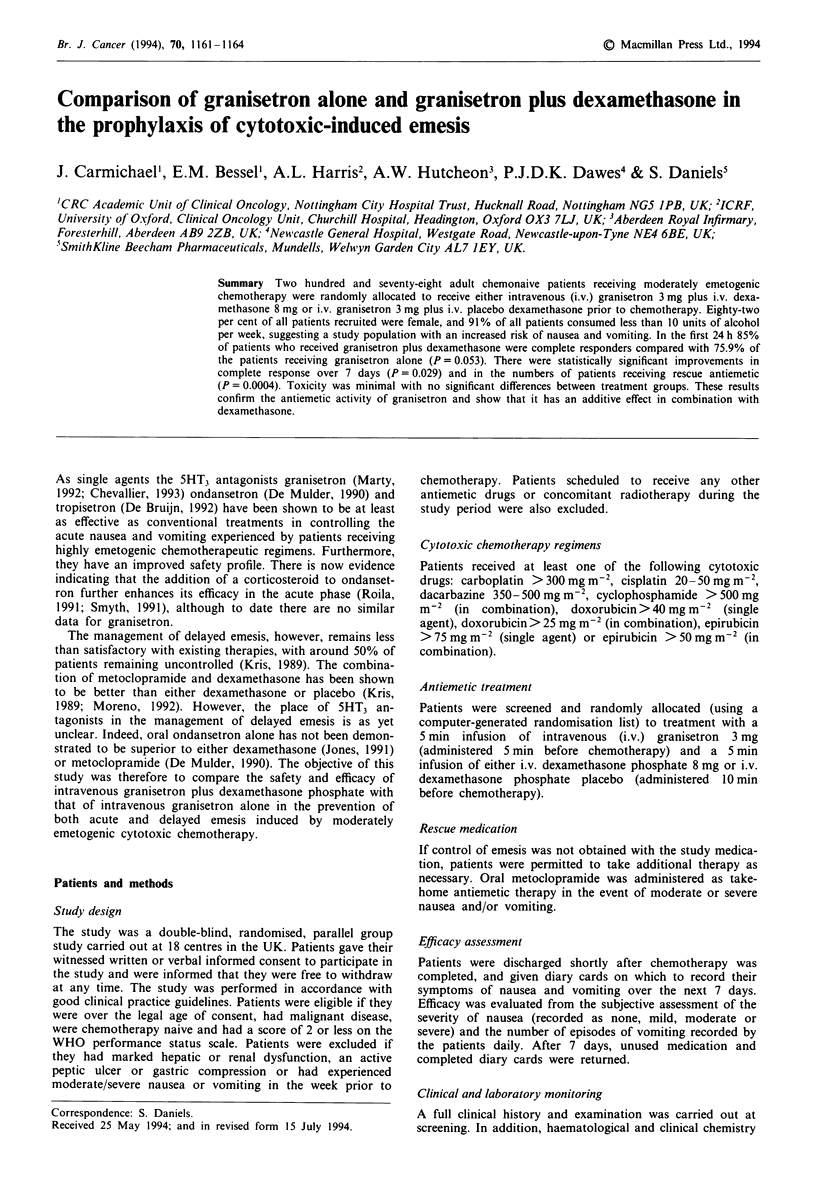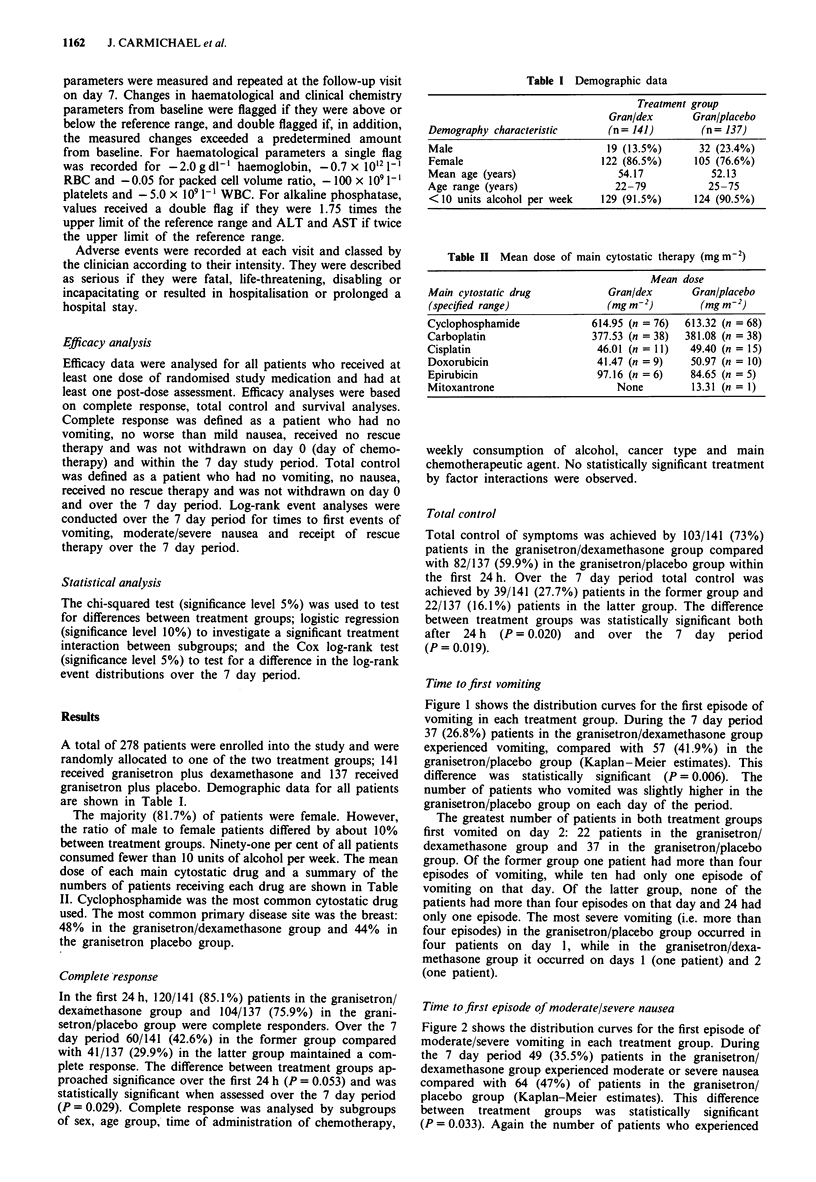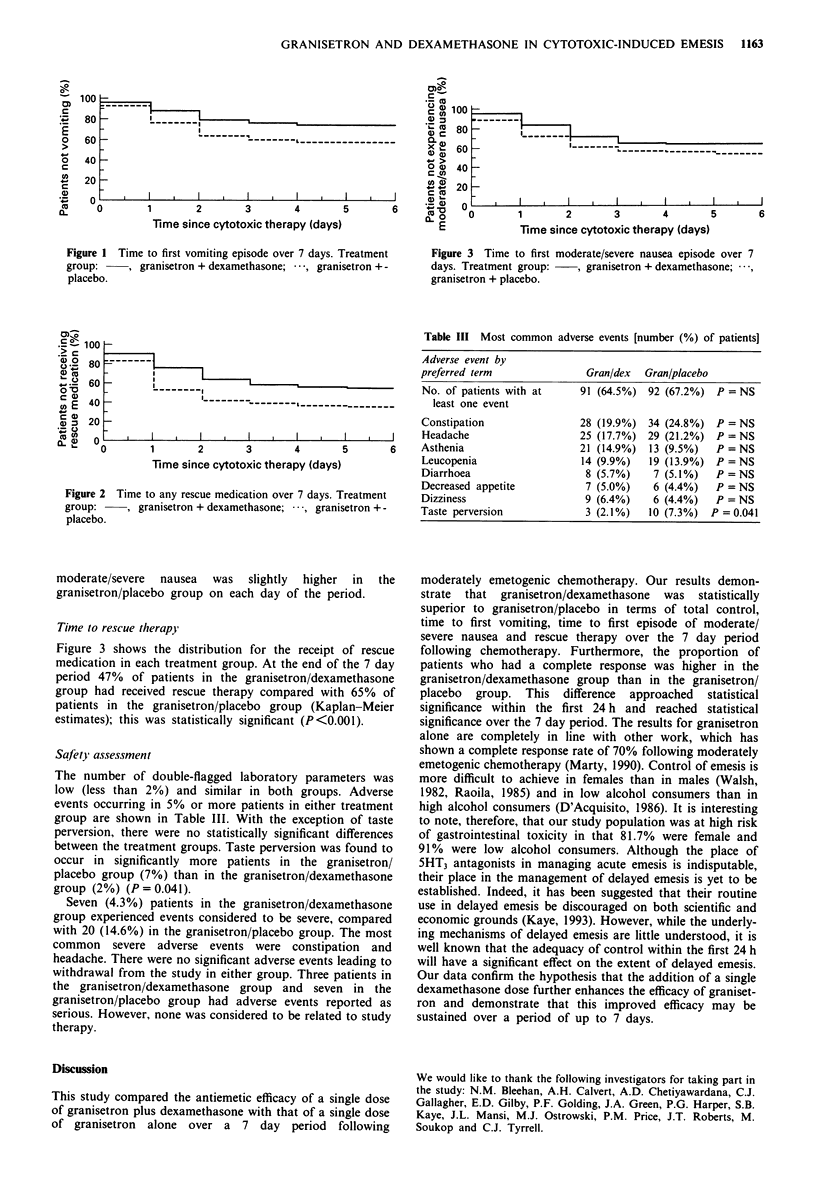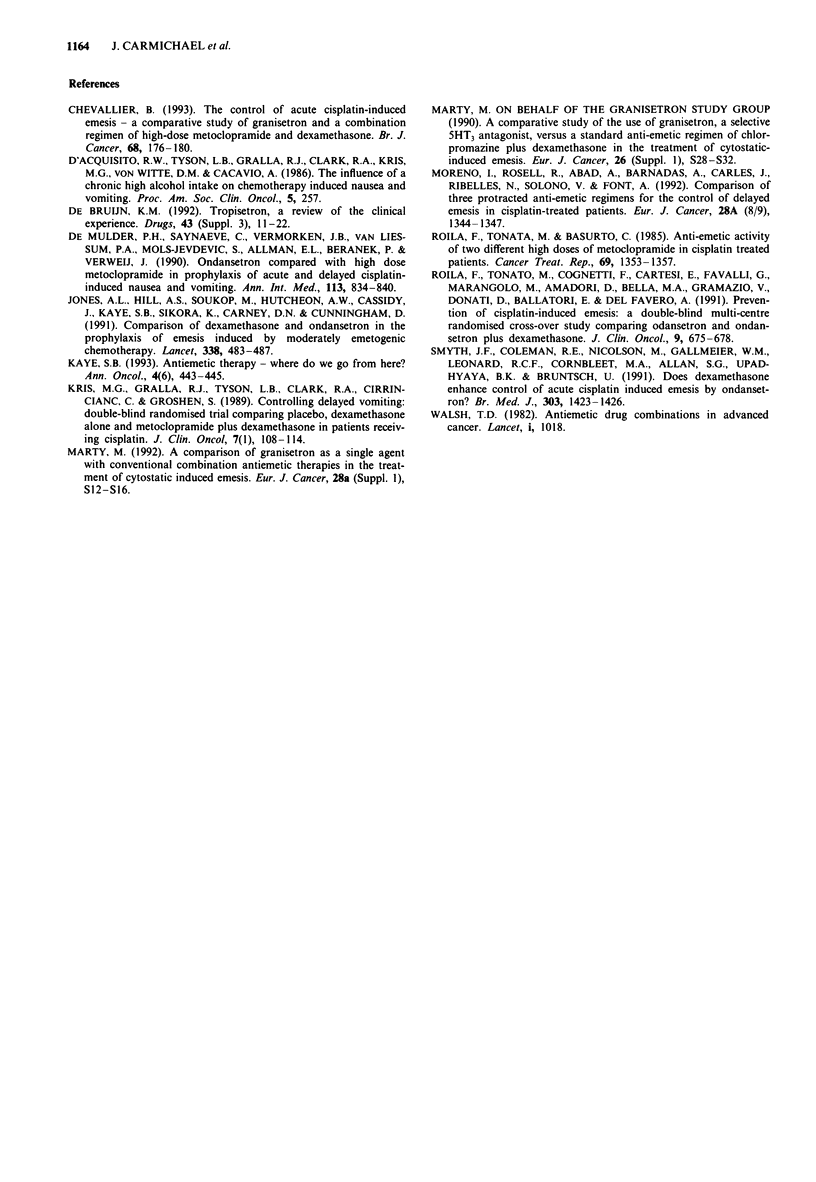# Comparison of granisetron alone and granisetron plus dexamethasone in the prophylaxis of cytotoxic-induced emesis.

**DOI:** 10.1038/bjc.1994.465

**Published:** 1994-12

**Authors:** J. Carmichael, E. M. Bessell, A. L. Harris, A. W. Hutcheon, P. J. Dawes, S. Daniels, E. M. Bessel

**Affiliations:** CRC Academic Unit of Clinical Oncology, Nottingham City Hospital Trust, UK.

## Abstract

Two hundred and seventy-eight adult chemonaive patients, receiving moderately emetogenic chemotherapy were randomly allocated to receive either intravenous (i.v.) granisetron 3 mg plus i.v. dexamethasone 8 mg or i.v. granisetron 3 mg plus i.v. placebo dexamethasone prior to chemotherapy. Eight-two per cent of all patients recruited were female, and 91% of all patients consumed less than 10 units of alcohol per week, suggesting a study population with an increased risk of nausea and vomiting. In the first 24 h 85% of patients who received granisetron plus dexamethasone were complete responders compared with 75.9% of the patients receiving granisetron alone (P = 0.053). There were statistically significant improvements in complete response over 7 days (P = 0.029) and in the numbers of patients receiving rescue antiemetic (P = 0.0004). Toxicity was minimal with no significant differences between treatment groups. These results confirm the antiemetic activity of granisetron and show that it has an additive effect in combination with dexamethasone.


Br. J. Cancer (1994), 70, 1161–1164 © Macmillan Press Ltd., 1994

### Comparison of granisetron alone and granisetron plus dexamethasone in the prophylaxis of cytotoxic-induced emesis

J. Carmichael[1], E.M. Bessel[1], A.L. Harris[2], A.W. Hutcheon[3], P.J.D.K. Dawes[4] & S. Daniels[5]

[1]*CRC Academic Unit of Clinical Oncology, Nottingham City Hospital Trust, Hucknall Road, Nottingham NG5 1PB, UK;* [2]*ICRF, University of Oxford, Clinical Oncology Unit, Churchill Hospital, Headington, Oxford OX3 7LJ, UK;* [3]*Aberdeen Royal Infirmary, Foresterhill, Aberdeen AB9 2ZB, UK;* [4]*Newcastle General Hospital, Westgate Road, Newcastle-upon-Tyne NE4 6BE, UK;* [5]*SmithKline Beecham Pharmaceuticals, Mundells, Welwyn Garden City AL7 1EY, UK.*

**Summary** Two hundred and seventy-eight adult chemonaive patients receiving moderately emetogenic chemotherapy were randomly allocated to receive either intravenous (i.v.) granisetron 3 mg plus i.v. dexamethasone 8 mg or i.v. granisetron 3 mg plus i.v. placebo dexamethasone prior to chemotherapy. Eighty-two per cent of all patients recruited were female, and 91% of all patients consumed less than 10 units of alcohol per week, suggesting a study population with an increased risk of nausea and vomiting. In the first 24 h 85% of patients who received granisetron plus dexamethasone were complete responders compared with 75.9% of the patients receiving granisetron alone ($P = 0.053$). There were statistically significant improvements in complete response over 7 days ($P = 0.029$) and in the numbers of patients receiving rescue antiemetic ($P = 0.0004$). Toxicity was minimal with no significant differences between treatment groups. These results confirm the antiemetic activity of granisetron and show that it has an additive effect in combination with dexamethasone.

As single agents the $5HT_3$ antagonists granisetron (Marty, 1992; Chevallier, 1993) ondansetron (De Mulder, 1990) and tropisetron (De Bruijn, 1992) have been shown to be at least as effective as conventional treatments in controlling the acute nausea and vomiting experienced by patients receiving highly emetogenic chemotherapeutic regimens. Furthermore, they have an improved safety profile. There is now evidence indicating that the addition of a corticosteroid to ondansetron further enhances its efficacy in the acute phase (Roila, 1991; Smyth, 1991), although to date there are no similar data for granisetron.

The management of delayed emesis, however, remains less than satisfactory with existing therapies, with around 50% of patients remaining uncontrolled (Kris, 1989). The combination of metoclopramide and dexamethasone has been shown to be better than either dexamethasone or placebo (Kris, 1989; Moreno, 1992). However, the place of $5HT_3$ antagonists in the management of delayed emesis is as yet unclear. Indeed, oral ondansetron alone has not been demonstrated to be superior to either dexamethasone (Jones, 1991) or metoclopramide (De Mulder, 1990). The objective of this study was therefore to compare the safety and efficacy of intravenous granisetron plus dexamethasone phosphate with that of intravenous granisetron alone in the prevention of both acute and delayed emesis induced by moderately emetogenic cytotoxic chemotherapy.

### Patients and methods

### *Study design*

The study was a double-blind, randomised, parallel group study carried out at 18 centres in the UK. Patients gave their witnessed written or verbal informed consent to participate in the study and were informed that they were free to withdraw at any time. The study was performed in accordance with good clinical practice guidelines. Patients were eligible if they were over the legal age of consent, had malignant disease, were chemotherapy naive and had a score of 2 or less on the WHO performance status scale. Patients were excluded if they had marked hepatic or renal dysfunction, an active peptic ulcer or gastric compression or had experienced moderate/severe nausea or vomiting in the week prior to chemotherapy. Patients scheduled to receive any other antiemetic drugs or concomitant radiotherapy during the study period were also excluded.

### *Cytotoxic chemotherapy regimens*

Patients received at least one of the following cytotoxic drugs: carboplatin $>300\ mg\ m^{-2}$, cisplatin $20-50\ mg\ m^{-2}$, dacarbazine $350-500\ mg\ m^{-2}$, cyclophosphamide $>500\ mg\ m^{-2}$ (in combination), doxorubicin $>40\ mg\ m^{-2}$ (single agent), doxorubicin $>25\ mg\ m^{-2}$ (in combination), epirubicin $>75\ mg\ m^{-2}$ (single agent) or epirubicin $>50\ mg\ m^{-2}$ (in combination).

### *Antiemetic treatment*

Patients were screened and randomly allocated (using a computer-generated randomisation list) to treatment with a 5 min infusion of intravenous (i.v.) granisetron 3 mg (administered 5 min before chemotherapy) and a 5 min infusion of either i.v. dexamethasone phosphate 8 mg or i.v. dexamethasone phosphate placebo (administered 10 min before chemotherapy).

### *Rescue medication*

If control of emesis was not obtained with the study medication, patients were permitted to take additional therapy as necessary. Oral metoclopramide was administered as take-home antiemetic therapy in the event of moderate or severe nausea and/or vomiting.

### *Efficacy assessment*

Patients were discharged shortly after chemotherapy was completed, and given diary cards on which to record their symptoms of nausea and vomiting over the next 7 days. Efficacy was evaluated from the subjective assessment of the severity of nausea (recorded as none, mild, moderate or severe) and the number of episodes of vomiting recorded by the patients daily. After 7 days, unused medication and completed diary cards were returned.

### *Clinical and laboratory monitoring*

A full clinical history and examination was carried out at screening. In addition, haematological and clinical chemistry

Correspondence: S. Daniels.
Received 25 May 1994; and in revised form 15 July 1994.

parameters were measured and repeated at the follow-up visit on day 7. Changes in haematological and clinical chemistry parameters from baseline were flagged if they were above or below the reference range, and double flagged if, in addition, the measured changes exceeded a predetermined amount from baseline. For haematological parameters a single flag was recorded for $-2.0\ g\ dl^{-1}$ haemoglobin, $-0.7 \times 10^{12}\ l^{-1}$ RBC and $-0.05$ for packed cell volume ratio, $-100 \times 10^{9}\ l^{-1}$ platelets and $-5.0 \times 10^{9}\ l^{-1}$ WBC. For alkaline phosphatase, values received a double flag if they were 1.75 times the upper limit of the reference range and ALT and AST if twice the upper limit of the reference range.

Adverse events were recorded at each visit and classed by the clinician according to their intensity. They were described as serious if they were fatal, life-threatening, disabling or incapacitating or resulted in hospitalisation or prolonged a hospital stay.

### *Efficacy analysis*

Efficacy data were analysed for all patients who received at least one dose of randomised study medication and had at least one post-dose assessment. Efficacy analyses were based on complete response, total control and survival analyses. Complete response was defined as a patient who had no vomiting, no worse than mild nausea, received no rescue therapy and was not withdrawn on day 0 (day of chemotherapy) and within the 7 day study period. Total control was defined as a patient who had no vomiting, no nausea, received no rescue therapy and was not withdrawn on day 0 and over the 7 day period. Log-rank event analyses were conducted over the 7 day period for times to first events of vomiting, moderate/severe nausea and receipt of rescue therapy over the 7 day period.

### *Statistical analysis*

The chi-squared test (significance level 5%) was used to test for differences between treatment groups; logistic regression (significance level 10%) to investigate a significant treatment interaction between subgroups; and the Cox log-rank test (significance level 5%) to test for a difference in the log-rank event distributions over the 7 day period.

### Results

A total of 278 patients were enrolled into the study and were randomly allocated to one of the two treatment groups; 141 received granisetron plus dexamethasone and 137 received granisetron plus placebo. Demographic data for all patients are shown in Table I.

The majority (81.7%) of patients were female. However, the ratio of male to female patients differed by about 10% between treatment groups. Ninety-one per cent of all patients consumed fewer than 10 units of alcohol per week. The mean dose of each main cytostatic drug and a summary of the numbers of patients receiving each drug are shown in Table II. Cyclophosphamide was the most common cytostatic drug used. The most common primary disease site was the breast: 48% in the granisetron/dexamethasone group and 44% in the granisetron placebo group.

### *Complete response*

In the first 24 h, 120/141 (85.1%) patients in the granisetron/dexamethasone group and 104/137 (75.9%) in the granisetron/placebo group were complete responders. Over the 7 day period 60/141 (42.6%) in the former group compared with 41/137 (29.9%) in the latter group maintained a complete response. The difference between treatment groups approached significance over the first 24 h ($P = 0.053$) and was statistically significant when assessed over the 7 day period ($P = 0.029$). Complete response was analysed by subgroups of sex, age group, time of administration of chemotherapy, weekly consumption of alcohol, cancer type and main chemotherapeutic agent. No statistically significant treatment by factor interactions were observed.

**Table I** Demographic data

| Demography characteristic | Treatment group<br>Gran/dex (n = 141) | Gran/placebo (n = 137) |
|---|---|---|
| Male | 19 (13.5%) | 32 (23.4%) |
| Female | 122 (86.5%) | 105 (76.6%) |
| Mean age (years) | 54.17 | 52.13 |
| Age range (years) | 22–79 | 25–75 |
| <10 units alcohol per week | 129 (91.5%) | 124 (90.5%) |

**Table II** Mean dose of main cytostatic therapy ($mg\ m^{-2}$)

| Main cytostatic drug (specified range) | Mean dose<br>Gran/dex ($mg\ m^{-2}$) | Gran/placebo ($mg\ m^{-2}$) |
|---|---|---|
| Cyclophosphamide | 614.95 ($n = 76$) | 613.32 ($n = 68$) |
| Carboplatin | 377.53 ($n = 38$) | 381.08 ($n = 38$) |
| Cisplatin | 46.01 ($n = 11$) | 49.40 ($n = 15$) |
| Doxorubicin | 41.47 ($n = 9$) | 50.97 ($n = 10$) |
| Epirubicin | 97.16 ($n = 6$) | 84.65 ($n = 5$) |
| Mitoxantrone | None | 13.31 ($n = 1$) |

### *Total control*

Total control of symptoms was achieved by 103/141 (73%) patients in the granisetron/dexamethasone group compared with 82/137 (59.9%) in the granisetron/placebo group within the first 24 h. Over the 7 day period total control was achieved by 39/141 (27.7%) patients in the former group and 22/137 (16.1%) patients in the latter group. The difference between treatment groups was statistically significant both after 24 h ($P = 0.020$) and over the 7 day period ($P = 0.019$).

### *Time to first vomiting*

Figure 1 shows the distribution curves for the first episode of vomiting in each treatment group. During the 7 day period 37 (26.8%) patients in the granisetron/dexamethasone group experienced vomiting, compared with 57 (41.9%) in the granisetron/placebo group (Kaplan–Meier estimates). This difference was statistically significant ($P = 0.006$). The number of patients who vomited was slightly higher in the granisetron/placebo group on each day of the period.

The greatest number of patients in both treatment groups first vomited on day 2: 22 patients in the granisetron/dexamethasone group and 37 in the granisetron/placebo group. Of the former group one patient had more than four episodes of vomiting, while ten had only one episode of vomiting on that day. Of the latter group, none of the patients had more than four episodes on that day and 24 had only one episode. The most severe vomiting (i.e. more than four episodes) in the granisetron/placebo group occurred in four patients on day 1, while in the granisetron/dexamethasone group it occurred on days 1 (one patient) and 2 (one patient).

### *Time to first episode of moderate/severe nausea*

Figure 2 shows the distribution curves for the first episode of moderate/severe vomiting in each treatment group. During the 7 day period 49 (35.5%) patients in the granisetron/dexamethasone group experienced moderate or severe nausea compared with 64 (47%) of patients in the granisetron/placebo group (Kaplan–Meier estimates). This difference between treatment groups was statistically significant ($P = 0.033$). Again the number of patients who experienced

**Figure 1** Time to first vomiting episode over 7 days. Treatment group: ——, granisetron + dexamethasone; ···, granisetron + placebo.

**Figure 3** Time to first moderate/severe nausea episode over 7 days. Treatment group: ——, granisetron + dexamethasone; ···, granisetron + placebo.

**Figure 2** Time to any rescue medication over 7 days. Treatment group: ——, granisetron + dexamethasone; ···, granisetron + placebo.

**Table III** Most common adverse events [number (%) of patients]

| *Adverse event by preferred term* | *Gran/dex* | *Gran/placebo* | |
|---|---|---|---|
| No. of patients with at least one event | 91 (64.5%) | 92 (67.2%) | $P$ = NS |
| Constipation | 28 (19.9%) | 34 (24.8%) | $P$ = NS |
| Headache | 25 (17.7%) | 29 (21.2%) | $P$ = NS |
| Asthenia | 21 (14.9%) | 13 (9.5%) | $P$ = NS |
| Leucopenia | 14 (9.9%) | 19 (13.9%) | $P$ = NS |
| Diarrhoea | 8 (5.7%) | 7 (5.1%) | $P$ = NS |
| Decreased appetite | 7 (5.0%) | 6 (4.4%) | $P$ = NS |
| Dizziness | 9 (6.4%) | 6 (4.4%) | $P$ = NS |
| Taste perversion | 3 (2.1%) | 10 (7.3%) | $P$ = 0.041 |

moderate/severe nausea was slightly higher in the granisetron/placebo group on each day of the period.

### *Time to rescue therapy*

Figure 3 shows the distribution for the receipt of rescue medication in each treatment group. At the end of the 7 day period 47% of patients in the granisetron/dexamethasone group had received rescue therapy compared with 65% of patients in the granisetron/placebo group (Kaplan–Meier estimates); this was statistically significant ($P<0.001$).

### *Safety assessment*

The number of double-flagged laboratory parameters was low (less than 2%) and similar in both groups. Adverse events occurring in 5% or more patients in either treatment group are shown in Table III. With the exception of taste perversion, there were no statistically significant differences between the treatment groups. Taste perversion was found to occur in significantly more patients in the granisetron/placebo group (7%) than in the granisetron/dexamethasone group (2%) ($P = 0.041$).

Seven (4.3%) patients in the granisetron/dexamethasone group experienced events considered to be severe, compared with 20 (14.6%) in the granisetron/placebo group. The most common severe adverse events were constipation and headache. There were no significant adverse events leading to withdrawal from the study in either group. Three patients in the granisetron/dexamethasone group and seven in the granisetron/placebo group had adverse events reported as serious. However, none was considered to be related to study therapy.

### Discussion

This study compared the antiemetic efficacy of a single dose of granisetron plus dexamethasone with that of a single dose of granisetron alone over a 7 day period following moderately emetogenic chemotherapy. Our results demonstrate that granisetron/dexamethasone was statistically superior to granisetron/placebo in terms of total control, time to first vomiting, time to first episode of moderate/severe nausea and rescue therapy over the 7 day period following chemotherapy. Furthermore, the proportion of patients who had a complete response was higher in the granisetron/dexamethasone group than in the granisetron/placebo group. This difference approached statistical significance within the first 24 h and reached statistical significance over the 7 day period. The results for granisetron alone are completely in line with other work, which has shown a complete response rate of 70% following moderately emetogenic chemotherapy (Marty, 1990). Control of emesis is more difficult to achieve in females than in males (Walsh, 1982, Raoila, 1985) and in low alcohol consumers than in high alcohol consumers (D'Acquisito, 1986). It is interesting to note, therefore, that our study population was at high risk of gastrointestinal toxicity in that 81.7% were female and 91% were low alcohol consumers. Although the place of $5HT_3$ antagonists in managing acute emesis is indisputable, their place in the management of delayed emesis is yet to be established. Indeed, it has been suggested that their routine use in delayed emesis be discouraged on both scientific and economic grounds (Kaye, 1993). However, while the underlying mechanisms of delayed emesis are little understood, it is well known that the adequacy of control within the first 24 h will have a significant effect on the extent of delayed emesis. Our data confirm the hypothesis that the addition of a single dexamethasone dose further enhances the efficacy of granisetron and demonstrate that this improved efficacy may be sustained over a period of up to 7 days.

We would like to thank the following investigators for taking part in the study: N.M. Bleehan, A.H. Calvert, A.D. Chetiyawardana, C.J. Gallagher, E.D. Gilby, P.F. Golding, J.A. Green, P.G. Harper, S.B. Kaye, J.L. Mansi, M.J. Ostrowski, P.M. Price, J.T. Roberts, M. Soukop and C.J. Tyrrell.

### References

CHEVALLIER, B. (1993). The control of acute cisplatin-induced emesis – a comparative study of granisetron and a combination regimen of high-dose metoclopramide and dexamethasone. *Br. J. Cancer*, **68**, 176–180.

D'ACQUISITO, R.W., TYSON, L.B., GRALLA, R.J., CLARK, R.A., KRIS, M.G., VON WITTE, D.M. & CACAVIO, A. (1986). The influence of a chronic high alcohol intake on chemotherapy induced nausea and vomiting. *Proc. Am. Soc. Clin. Oncol.*, **5**, 257.

DE BRUIJN, K.M. (1992). Tropisetron, a review of the clinical experience. *Drugs*, **43** (Suppl. 3), 11–22.

DE MULDER, P.H., SAYNAEVE, C., VERMORKEN, J.B., VAN LIESSUM, P.A., MOLS-JEVDEVIC, S., ALLMAN, E.L., BERANEK, P. & VERWEIJ, J. (1990). Ondansetron compared with high dose metoclopramide in prophylaxis of acute and delayed cisplatin-induced nausea and vomiting. *Ann. Int. Med.*, **113**, 834–840.

JONES, A.L., HILL, A.S., SOUKOP, M., HUTCHEON, A.W., CASSIDY, J., KAYE, S.B., SIKORA, K., CARNEY, D.N. & CUNNINGHAM, D. (1991). Comparison of dexamethasone and ondansetron in the prophylaxis of emesis induced by moderately emetogenic chemotherapy. *Lancet*, **338**, 483–487.

KAYE, S.B. (1993). Antiemetic therapy – where do we go from here? *Ann. Oncol.*, **4**(6), 443–445.

KRIS, M.G., GRALLA, R.J., TYSON, L.B., CLARK, R.A., CIRRINCIANC, C. & GROSHEN, S. (1989). Controlling delayed vomiting: double-blind randomised trial comparing placebo, dexamethasone alone and metoclopramide plus dexamethasone in patients receiving cisplatin. *J. Clin. Oncol*, **7**(1), 108–114.

MARTY, M. (1992). A comparison of granisetron as a single agent with conventional combination antiemetic therapies in the treatment of cytostatic induced emesis. *Eur. J. Cancer*, **28a** (Suppl. 1), S12–S16.

MARTY, M. ON BEHALF OF THE GRANISETRON STUDY GROUP (1990). A comparative study of the use of granisetron, a selective $5HT_3$ antagonist, versus a standard anti-emetic regimen of chlorpromazine plus dexamethasone in the treatment of cytostatic-induced emesis. *Eur. J. Cancer*, **26** (Suppl. 1), S28–S32.

MORENO, I., ROSELL, R., ABAD, A., BARNADAS, A., CARLES, J., RIBELLES, N., SOLONO, V. & FONT, A. (1992). Comparison of three protracted anti-emetic regimens for the control of delayed emesis in cisplatin-treated patients. *Eur. J. Cancer*, **28A** (8/9), 1344–1347.

ROILA, F., TONATA, M. & BASURTO, C. (1985). Anti-emetic activity of two different high doses of metoclopramide in cisplatin treated patients. *Cancer Treat. Rep.*, **69**, 1353–1357.

ROILA, F., TONATO, M., COGNETTI, F., CARTESI, E., FAVALLI, G., MARANGOLO, M., AMADORI, D., BELLA, M.A., GRAMAZIO, V., DONATI, D., BALLATORI, E. & DEL FAVERO, A. (1991). Prevention of cisplatin-induced emesis: a double-blind multi-centre randomised cross-over study comparing odansetron and ondansetron plus dexamethasone. *J. Clin. Oncol.*, **9**, 675–678.

SMYTH, J.F., COLEMAN, R.E., NICOLSON, M., GALLMEIER, W.M., LEONARD, R.C.F., CORNBLEET, M.A., ALLAN, S.G., UPADHYAYA, B.K. & BRUNTSCH, U. (1991). Does dexamethasone enhance control of acute cisplatin induced emesis by ondansetron? *Br. Med. J.*, **303**, 1423–1426.

WALSH, T.D. (1982). Antiemetic drug combinations in advanced cancer. *Lancet*, **i**, 1018.