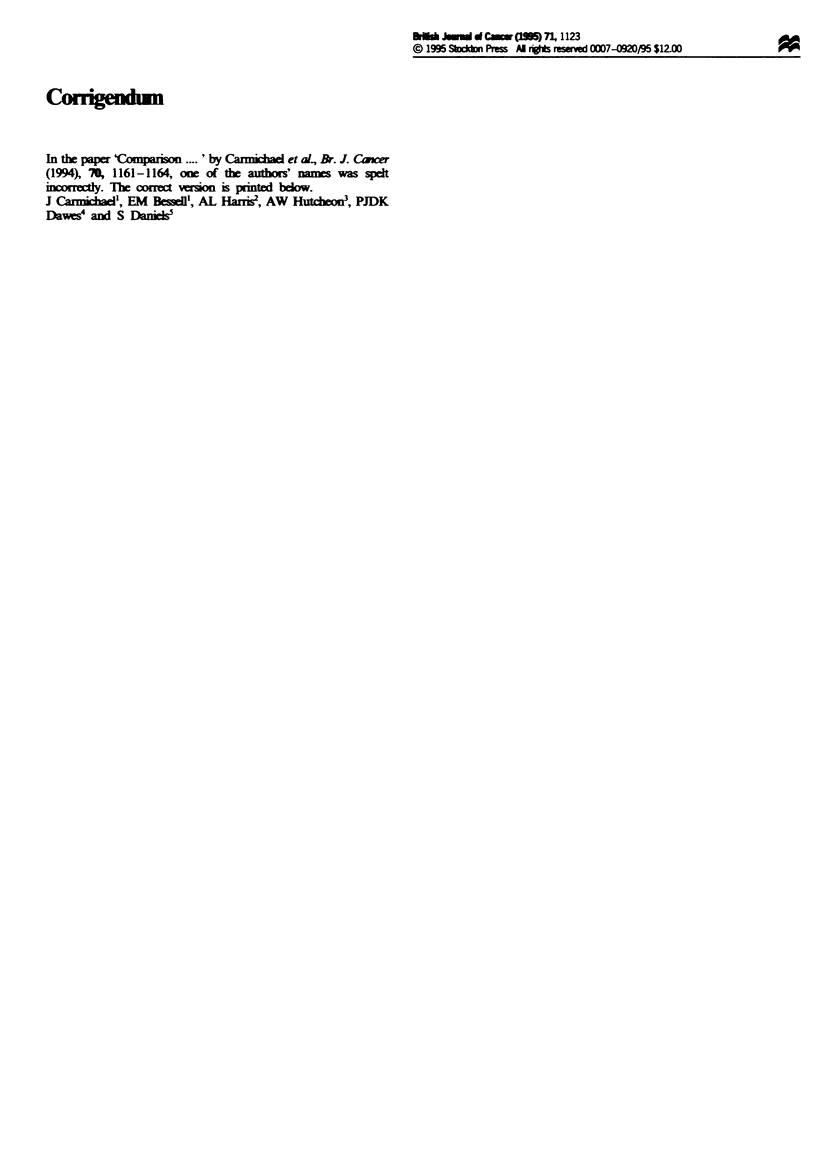# Corrigendum

**Published:** 1995-05

**Authors:** 


					
brlft Jsmi ud CwQSU9) 71, 1123

? 1995 S2don Press Al rhts reserwd 0007-0920/95 $12 00

In tbe pa     omparison .... ' by Carmichael et at, Br. J. Ceicer
(1994), 70, 1161-1164, one of the autiors' names was spelt
mxorewty. Tfl corre    vsson is primted below.

J Carm1hl', EM Bessel', AL Haffise, AW    Hutchoo3, PJDK
Dawes& and S DanieW